# Modern Immunotherapy of Adult B-Lineage Acute Lymphoblastic Leukemia with Monoclonal Antibodies and Chimeric Antigen Receptor Modified T Cells

**DOI:** 10.4084/MJHID.2015.001

**Published:** 2015-01-01

**Authors:** Elena Maino, Anna Maria Scattolin, Piera Viero, Rosaria Sancetta, Anna Pascarella, Michele Vespignani, Renato Bassan

**Affiliations:** 1 Hematology and Bone Marrow Transplant Unit, Ospedale dell’Angelo e SS. Giovanni e Paolo, Mestre-Venezia, Italy

## Abstract

The introduction of newer cytotoxic monoclonal antibodies and chimeric antigen receptor modified T cells is opening a new age in the management of B-lineage adult acute lymphoblastic leukemia. This therapeutic change must be very positively acknowledged because of the limits of intensive chemotherapy programs and allogeneic stem cell transplantation. In fact, with these traditional therapeutic tools the cure can be achieved in only 40–50% of the patients. The failure rates are particularly high in the elderly, in patients with post-induction persistence of minimal residual disease and especially in refractory/relapsed disease. The place of the novel immunotherapeutics in improving the outcome of adult patients with B-lineage acute lymphoblastic leukemia is reviewed.

## Introduction

Adult acute lymphoblastic leukemia (ALL) is biologically heterogeneous and can be subdivided into several clinico-prognostic entities.^1^ The primary distinction is between B-cell and T-cell precursor (BCP, TCP) ALL, and in the former group between Philadelphia chromosome/BCR-ABL (Ph) positive and Ph− ALL. The overall outcome of adults with ALL is inferior to that of childhood ALL. Basically, survival is strictly related to a complete remission (CR) achieved early on, which is followed by an effective consolidation/maintenance therapy, in standard-risk patients (SR) and, an allogeneic stem cell transplantation (SCT), in high-risk (HR) patients.^2^,^3^ In adolescent and adult patients with Ph− ALL in an age range between 15–18 and 60–65 years, the CR rate is 90% and, the overall survival (OS) rate is 40–50% at 3–5 years, with significant differences among age and risk groups.^4^,^5^ In Ph+ ALL, results are suboptimal too despite the improvement due to the introduction of tyrosine kinase inhibitors.^6^ In Ph− ALL, better OS, and disease-free survival (DFS) rates are increasingly reported using pediatric-inspired schedules, at least in patients aged up to 40–50 years.^7^ The outcome is worse in patients older than 55 years, with smaller proportions of long-term survivors.^8^ Moreover during CR induction about 5% of the patients succumb to early complications, mainly infectious, and the risk of non-relapse mortality is still rather high after an allogeneic SCT (15% on the average). Overall, the common perception is that treatment intensity cannot be increased any further beyond this point in adult patients, without incurring into unacceptably high rates of treatment-related toxicity and mortality. Instead, new alternative therapeutics should be developed with a view of reducing the toxicity burden other than improving the antileukemic efficacy of available antileukemic programs. In addition, the relapse rate in adult ALL remains high and salvage therapy is at present unsatisfactory, with an effective rescue rate of 10–20% in most studies.

The most recent therapeutic innovations are represented by newer monoclonal antibodies (MoAb) and the chimeric antigen receptor (CAR) modified T cells. These new, highly selective weapons target specific ALL cell antigens and would exhibit an improved activity versus toxicity ratio compared to chemotherapy or transplantation. In addition, they could be used sequentially or in combination with either treatment modality, to potentiate the overall treatment efficacy. Thus far, MoAb-based therapy and CAR T cell therapy were developed mainly for B-lineage but not T-lineage ALL. They have been utilized in all B-lineage subsets (BCP and mature B/Burkitt ALL; Ph− and Ph+ ALL), and demonstrated considerable activity in relapsed/refractory disease (R/R ALL). Therefore, they need to be exploited in untreated ALL, especially in high-risk subsets such as the elderly and the patient with high post-induction levels of minimal residual disease (MRD). Here we review the evidence supporting the use of therapeutic MoAb and CAR T cells in BCP ALL. Additional data can be found elsewhere.^9^–^11^ Results from childhood studies will be reported whenever appropriate to illustrate specific points of interest.

## Modern Immunotherapy with Monoclonal Antibodies

The challenge of novel immunotherapeutics is to improve survival without increasing toxicity. With MoAbs, the different and manageable toxicity profile only occasionally overlaps or worsens that associated with chemotherapy and SCT. For instance, mucositis and gastrointestinal toxicity, usually of high concern with intensive chemotherapy and SCT, are not typical of MoAb therapy. The apparent lack of cross-resistance with standard antileukemic drugs constitutes a further theoretic advantage. The third major issue is whether MoAb therapy might substitute, at least partially, for some intensive chemotherapy elements and/or SCT in patients in CR1. Prospective clinical trials should address this most important topic.

ALL cells express several membrane antigens. The ideal therapeutic target should be consistently expressed in every ALL subset, by all blast cells, at high intensity, be stable upon MoAb challenging and play a crucial role in metabolic events. At present, no MoAb satisfies all these requirements and a target expression of 20% out of the entire ALL cell population is considered enough to start a MoAb trial with some chance of success.

**Figure 1:**
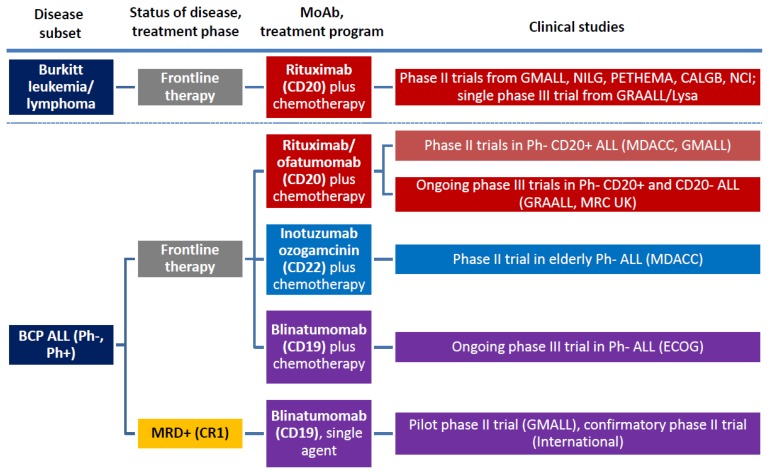
Overview of MoAb studies with rituximab, inotuzumab ozogamicin, andblinatumomab in frontline therapy of adult BCP ALL and Burkitt leukemia/lymphoma.

**Figure 2:**
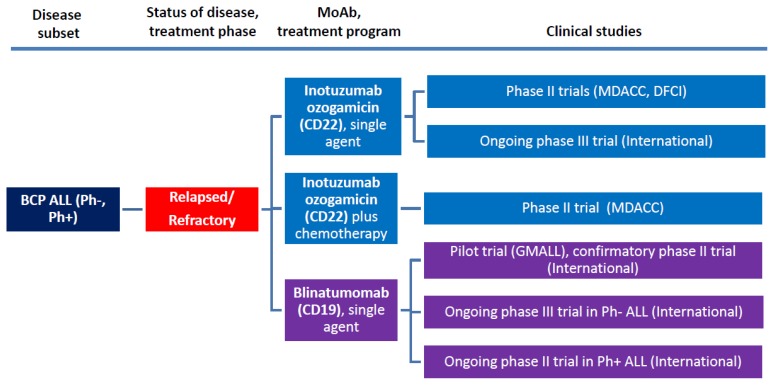
Overview of MoAb studies with rituximab, inotuzumab ozogamicin, and blinatumomab in adult R/R BCP ALL.

According to their structural characteristics and mechanism of action, MoAbs for ALL therapy belong to three major categories: naked antibodies, T-cell engaging bispecific single-chain (BiTE^®^) antibodies, and immunoconjugates/immunotoxins. The several trials launched with the most representative and therapeutically promising MoAb’s, with or without associated chemotherapy, are summarized in Figure 1 (frontline studies) and Figure 2 (studies in R/R ALL) and detailed below.

## Naked Antibodies

### Rituximab and Ofatumomab: anti-CD20 MoAb

The CD20 receptor is the target of chimeric monoclonal antibody Rituximab. CD20 is expressed by approximately 40% of BCP ALL cases and virtually any case of mature B-ALL (Burkitt leukemia). The CD20 receptor functions as a calcium channel playing a role in cell cycle and differentiation. Rituximab works as a classical MoAb, reacting at one terminus (Fab/Fv) with the CD20 epitope on the cell membrane, while the other end (Fc) binds to complement and Fc receptors of effectors cells. The ensuing MoAb-target cell interaction activates a complement-mediated cell lysis and/or an antibody-dependent cellular cytotoxicity (ADCC). Importantly, CD20 expression in CD20 + ALL is upregulated by corticosteroids, which are commonly given in prephase and continued for several days during induction therapy.^10^,^12^

Nothing is known about rituximab activity as a single agent in ALL, and contrary to other MoAbs experience in R/R ALL is very limited. One study indicated a response rate of 44% in 9 patients treated with a rituximab-chemotherapy combination.^13^

Rituximab was instead used in first-line phase II and III programs, and is used in Burkitt leukemia/lymphoma in adjunct to aggressive rotational drug regimens.

The usual rituximab schedule in these studies was 375 mg/m^2^ for four-eight times, throughout induction and consolidation blocks. A randomized trial in Burkitt lymphoma confirmed the usefulness of adding rituximab to intensive chemotherapy blocks, in both HIV negative and positive HIV patients.^14^,^15^ Several other Burkitt leukemia/lymphoma regimens reported high response rates, with a curability rate consistently above 50% and most often between 70–80% and close to 90%–100% in fit patients younger than 55–60 years.^14^–^21^ This means an average 20% or more improvement over prior results obtained with similar chemotherapy regimens without rituximab, with no substantial difference in toxic side effects. Nowadays rituximab is part of the standard of care for Burkitt leukemia/lymphoma.

About rituximab in frontline therapy of BCP ALL, there were two randomized trials and two phase II trials in Ph− ALL, all evaluating its role in addition to induction and consolidation chemotherapy. In the GRAALL (France/Belgium/Switzerland) phase III trial, CD20+ BCP, ALL patients (CD20 expression >20%) were randomized with a 2×2 design concurrently testing an augmented cyclophosphamide dose; whereas in the randomized MRC (United Kingdom) trial, all BCP ALL patients were randomized to assess the role of the concomitant corticosteroid therapy in upregulating CD20 expression in CD20− patients. The results from these two controlled studies are not yet known and are awaited with interest. As to phase II trials, in the MD Anderson Hospital study^22^ two sequential CD20+ BCP ALL patient cohorts receiving Hyper-CVAD chemotherapy with or without rituximab were analyzed. In patients aged 60 or less, the CR rate in the rituximab arm was 95% and 3-year survival 75% (n=68) compared with 47% without rituximab (n=46; P=0.003), with a proportional increase in MRD negativity evaluated by flow cytometry (81% vs 58%). A subsequent update showed for the rituximab-treated group a CR duration of 69% at 3 years with an OS of 71%.^23^ In the small group of patients older than 60 (n=16), the CR rate was high (88%) but the OS was only 29%. The other first-line phase II trial was from GMALL (Germany) with rituximab added to the 07/2003 chemotherapy schema.^24^ This report compared 181 rituximab-treated patients with 82 pre-rituximab patients. In SR patients (n=196), CR rate was 94% with rituximab and 91% without; however, minimal residual disease (MRD) response, evaluated molecularly at week 16 (<10^−4^) and, 5-year survival were both improved in the rituximab group, from 59% to 90% and from 57% to 71%, respectively. Similarly, in HR patients (n=67), CR rate was 81% with rituximab and 88% without; and MRD response and 5-year survival were improved from 40% to 64% and from 36% to 55%, respectively. Toxicities were comparable in the two cohorts. In summary, rituximab could improve the long-term outcome of patients with CD20+ BCP ALL and seems to enhance the MRD response to induction and early consideration therapy. This issue arises considerable interest, given the strict relationship between MRD and outcome in adult ALL and the dramatically worse outcome of MRD+ CD20+ ALL as opposed to MRD− CD20+ ALL.^25^ Although the CD20 antigen is expressed in a relevant proportion of Ph+ ALL cases, there is no data on the therapeutic role of this MoAb in this subset. The most significant data relative to the use of rituximab in B-lineage ALL Burkitt leukemia/lymphoma are summarized in Table 1.

Ofatumumab is another anti-CD20 MoAb, which binds to a different epitope on the CD20 molecule than rituximab, resulting in greater complement-dependent cytotoxicity. One study evaluated ofatumumab added to the Hyper-CVAD regimen as frontline therapy of adult patients with CD20+ ALL.^26^ With this regimen, 22 of 23 evaluable patients achieved CR (95%) and were MRD-negative (by flow cytometry) after cycle 1. One-year remission and OS duration was 91%.

### Epratuzumab: anti-CD22 MoAb

Epratuzumab is a humanized MoAb targeting CD22. The CD22 antigen is a transmembrane sialoglycoprotein expressed explicitly by B lymphoid cells. It is expressed on 100% of mature B-cell ALL and up to 90% of BCP ALL.^27^ CD22 regulates B-cell activation and the interaction of B-cells with T-cells and antigen-presenting cells. Because of that, CD22 is a good therapeutic target in BCP ALL. CD22 is rapidly internalized after binding the MoAb so that the exposure to epratuzumab results in downregulation of B-cell activation and signaling, with proliferation inhibition.^28^ In a phase I protocol ofthe Children’s Oncology Group (COG), applied to children with R/R BCP ALL, 15 children received four doses of epratuzumab twice weekly for two weeks, then four weekly doses with a standard reinduction chemotherapy. MRD was evaluated by flow cytometry, and the absence of MRD was defined as complete molecular remission (CMR). At the end of the six-week reinduction therapy, nine patients were in CR, and seven of them were in CMR. Two patients had dose-limiting toxicity, one grade four seizure, and one grade 3 transaminase elevation. A subsequent phase II trial (COG ADVL04P2)^28^,^29^ enrolled 114 patients between 2–30 years of age in first relapse, comparing two different epratuzumab schedules in addition to traditional reinduction chemotherapy. The CR rate was comparable in the two study arms (epratuzumab weekly × 4 doses versus epratuzumab twice weekly × 8 doses: CR 65% vs. 66%) and not significantly higher than the historical control. The CMR rate was however higher in epratuzumab-treated patients (42%) than historical controls.

The adult trial SWOG S0910^30^ evaluated 32 R/R ALL patients treated with epratuzumab (4 weekly doses) in association with clofarabine and cytarabine. The CR rate was 45%, significantly higher than the 17% CR rate observed in a similar trial with clofarabine/cytarabine without epratuzumab.^31^

Two other recent reports available only in abstract form concerned a phase I escalation study of ^90^yttrium-labeled epratuzumab tetratexan^32^ and epratuzumab added to vincristine/dexamethasone in R/R ALL.^33^ In the first study (n=17), 2 of six patients treated with a dose of 10 mCi/m^2^ achieved CR. In the second trial, including 26 elderly patients, four patients achieved CR, and one a CR with incomplete platelet recovery.

These are promising results obtained in very poor risk patient populations. Epratuzumab is well tolerated. The most common adverse events were myelosuppression and mild to moderate infusion reactions such as fever, nausea, occasionally seizures, and transaminase elevation.

### Alemtuzumab: anti-CD52 MoAb

Alemtuzumab is a genetically engineered humanized anti-CD52 MoAb. CD52 is a glycosylphosphatidylinositol-anchored membrane glycoprotein expressed by 70–80% of both BCP ALL and T-ALL, making it an attractive therapeutic target. Alemtuzumab has demonstrated significant activity in chronic lymphocytic leukemia but was not found effective as a single agent in acute myeloid leukemia and ALL.

In R/R ALL alemtuzumab was tested in a small adult series of 6 patients (3 with Ph+ ALL) at the dose of 30 mg given by subcutaneous route three times weekly for 4–12 weeks (no CR) and was also scarcely effective in a pediatric trial on 13 patients (one CR).^34^,^35^

In untreated patients, alemtuzumab was administered as a single agent in a CALGB trial^36^ after three intensive chemotherapy modules in an attempt to lower post-remission MRD. In 11 evaluable patients, there was a 1-log median MRD reduction and a noteworthy DFS (median 53 months), but follow-up was provided only for 14 surviving patients. Of note, the use of alemtuzumab was associated with CMV infection in 8 of 24 patients and herpes virus infection in 5 patients.

For these reasons, alemtuzumab, albeit partially effective, is unlikely to be developed any further in ALL therapy. It causes a drastic reduction of lymphocytes including CD4+ and CD8+ T cells predisposing to opportunistic infections such as CMV and other viruses and fungi.^36^ Thereafter, it requires careful patient monitoring with serial CMV DNA determinations for pre-emptive therapy, as well as an adequate anti-infectious prophylaxis.

## Immunotoxins and Immunoconjugate Antibodies

### Inotuzumab Ozogamicin: anti-CD22 MoAb

Inotuzumab ozogamicin (IO) is an anti-CD22 MoAb conjugated to calicheamicin, which is a powerful anthracycline-like drug. Calicheamicin, a natural product of Micromonospora echinospora,^37^ is a potent cytotoxic agent enabling cell killing even in the presence of relatively few target sites. Although CD22 expression is required, IO-related apoptotic effect is entirely mediated by calicheamicin and not by CD22 signaling. IO is rapidly internalized and delivers calicheamicin intracellularly. The toxin binds the minor DNA groove breaking the double-stranded DNA in a sequence-specific manner.

Forty-nine patients with R/R ALL were treated in a phase I/II trial at MD Anderson Hospital with single agent IO.^38^ Their median age was 36 years and range 6–80 years. All patients had greater that 50% CD22 expression on lymphoblasts, and the majority were heavily pretreated. A starting dose of 1.3 mg/m^2^ was used, subsequently increased to 1.8 mg/m^2^. The CR rate was 18% and another 39% of the patients had a CR with incomplete hematologic recovery (CRi), for an overall response rate of 57%. Among the 27 patients who achieved a hematological response, 17 (63%) attained an MRD remission (flow cytometry). Median response duration was six months, with a trend to improved survival for the 13 patients treated at first salvage. This study, updated including 90 total patients, confirmed the previous results (CR 19%, CRi 39%); furthermore, the non-hematological toxicity was reduced using the weekly schedule.^39^ Thus, with IO a morphological CR was obtained in more than 50% of the subjects treated, in association with a complete MRD response in the majority of these cases. Most responses were short lived without proceeding to transplantation (n=36), however the obtaining a CR with associated MRD response, the absence of a complex karyotype such as t(4;11), t(9;22), or an abnormal chromosome 17 and a disease status at first salvage were predictive of an improved outcome with a survival probability of 42+ months.^40^ A negative MRD was observed in 72% of the patients achieving CR/CRi. A new trial for R/R ALL incorporated IO into a reduced intensity Hyper-CVAD regimen.^41^ Of 35 patients treated, 18 (51%) entered CR, 6 (17%) CRi and 1 (3%) marrow CR, and 12 of them could proceed to allogeneic SCT. Median survival of responders was 14 months and was not reached in patients at first salvage. The outcome of IO-treated patients proceeding to allogeneic SCT was examined separately.^42^ The study analyzed the outcome of 26 such patients, of whom 23 were in CR at time of transplant (15 MRD-negative) and three were not. MRD-negative patients had the best outcome with a 1-year survival of 42%. However, non-relapse mortality was high in relation to liver toxicity (40% at six months), with 5 deaths by venoocclusive disease. These results could improve choosing the less hepatotoxic conditioning regimens and concomitant drugs. In conclusion, these single-center studies IO brought more patients with R/R ALL to allotransplantation (45%) than chemotherapy, but the salvage rate was affected by transplant-related toxicity, indicating the need of a careful design of all treatment components. An international phase III study comparing IO with standard reinduction therapy in R/R ALL is near to a conclusion.

In untreated patients, IO was added to mini-hyper-CVAD (dose reductions and no anthracycline) in elderly ALL.^43^ Twenty-seven patients aged 60–79 years (median 69 years) were treated, and 25 (96%) entered CR, all with negative flow cytometry MRD. The 1-year survival was 81%, superior to the historical control group. Although the follow-up is short, these are outstanding induction results obtained in a high-risk patient population. Another US Intergroup trial is planned in patients aged 18–39 years, adding IO to the C10403 chemotherapy backbone.

On the toxicity side, IO is myelotoxic, as reflected by the high rates of CRi. Grade 3–4 non-hematologic adverse events included drug-related fever (18%) with hypotension, hyperbilirubinemia (4%) and transaminase elevation (1%). All the events but the increased bilirubin were reversible. A biopsy demonstrated liver fibrosis in two patients. A venoocclusive disease of the liver was reported in 5/22 patients after allogeneic SCT.^39^ However, 4 of 5 of these patients received a preparative regimen of clofarabine/thiotepa. Furthermore, two distinct reports suggest a benefit toward liver toxicity with weekly rather than single dose IO administration.^39^,^44^

The most significant data relative to the use of IO in B-lineage ALL are summarized in Table 2.

### BL22 and CAT-8015: anti-CD22 MoAbs

Because the CD22 antigen-immunotoxins is rapidly internalized, CD22 is an attractive therapeutic target.^45^ The first-generation immunotoxins BL22 demonstrated cytotoxicity in vitro and also in vivo and in a phase 1 trial. A decrease of leukemia blasts was observed in 16 out 23 ALL patients, but no CR was obtained.^46^ Three of these patients developed neutralizing antibodies,^47^ but no allergic reaction, vascular leak or hemolytic uremic syndrome occurred.

A second-generation immunotoxins, CAT-8015, was subsequently developed,^45^ trying to reduce non-specific toxicities, increase MoAb stability and improve activity.^46^ In one small trial, 4 out of 9 treated patients achieved a CR.^10^ Another phase I trial showed a CR in 4 out of 19 heavily pretreated children and young adults, plus one partial response and 8 hematological improvements.^47^ Resistance due to low levels of DPH4 mRNA and target protein was described.^48^ Further analysis of the DPH4 gene promoter demonstrated hypermethylation in the resistant cells. This mechanism could be reversed by hypomethylating agents such as 5-azacytidine.

### Combotox: dual anti-CD19/CD22 MoAb

Combotox is a combination of anti-CD19 and anti-CD22 deglycosylated ricin-A chain immunotoxin.^49^ This treatment has the advantage of targeting two different antigens. In a pediatric trial, 3 of 17 R/R patients achieved a CR.^50^ The dose-limiting toxicity was a vascular leak syndrome, caused by an endothelial damage due to a unique aminoacid motif in the ricin-A toxin. Preclinical studies in murine ALL model demonstrated synergy with the sequential administration of combotox with cytarabine.

### SAR3419 and anti-B4-blocked ricin: anti-CD19 MoAb

SAR3419 is an anti-CD19 humanized MoAb linked to a highly powerful tubulin inhibitor, maytansinoid DM4, eliciting ADCC.^51^ SAR3419 is internalized and then routed to lysosomes, whereupon it is degraded to yield the active drug. In preclinical models, an extended duration of remission was documented when SAR3419 was administered after an induction regimen as maintenance therapy.^52^ A Phase II trial on R/R ALL is ongoing. Reversible corneal toxicity was described as dose-limiting toxicity.

The anti-B4-blocked ricin MoAb was used in a frontline CALGB study in patients with CD19+ ALL instead of high-dose cytarabine consolidation, reserved to CD19-negative ALL patients.^53^ Forty-six patients were treated. Although feasible, this treatment did not result into an improved outcome and/or MRD response compared to the other patients.

## Bispecific T-Cell Engager (BiTE^®^) Antibodies

### Blinatumomab: anti-CD3/CD19 construct

Blinatumomab is the first member of the novel class of BiTE^®^ antibodies. It is a bispecific single-chain antibody construct which simultaneously reacts to CD19 and CD3 epitopes, activating CD3+ T cells and re-directing their cytotoxicity against CD19+ ALL cells. Activated T cells induce perforin-mediated death on the target cells.^54^ CD19 is the most commonly expressed antigen in BCP ALL, with the highest density of expression and a slower internalization rate compared with CD22. Blinatumomab is given by continuous intravenous infusion at nine μg/d on days 1–7 and 28 μg/d on days 8–28, using a portable infusion device. A two-week interval follows each cycle. Although blinatumomab is active at very low concentrations, the prolonged infusion is necessary to recruit and expand effector T-cells and achieve therapeutic efficacy in the bone marrow.^55^

The first pilot trial was conducted in MRD+ ALL.^54^,^56^ MRD+ ALL is a high-risk condition, recognized by the persistence of the molecular signal of the disease in remission marrows after induction-consolidation therapy, usually, at a level of 10^−4^ or, greater after induction/early consolidation therapy, or by the reappearance of the MRD signal during follow up.^57^ MRD positivity heralds the clinical relapse within few weeks or months, but beside that, it is a more favorable setting than R/R ALL, because MRD+ patients still exhibit a good performance status and harbor a significantly lower disease burden. Blinatumomab was administered to 21 MRD+ patients as a four-week continuous infusion; the median patient age was 47 years and 7 patients had poor-risk cytogenetics (5 Ph+ and 2 with mixed lineage leukemia). Ten of 20 evaluable patients achieved a major MRD response <10^−4^, including 3 of 5 Ph+ ALL (60%). Most notably, 9 out of 11 patients with an MRD >10^−2^ achieved a molecular remission and 6 out of 11 not having a subsequent allogeneic SCT remained in CR after a median follow-up of 30 months, compared to 6 of 9 patients receiving an allogeneic SCT. Treatment toxicity consisted of an early cytokine release syndrome (pyrexia, chills), plus increased transaminases, albumin reduction, hypokalemia and an acute neurological syndrome (seizure, syncope, headache, somnolence) which was reversible in all cases. Due to these encouraging results a larger confirmatory phase II trial was performed (n=116), with 106 patients evaluable in an early report.^58^ Rates of complete MRD response were 78% after one cycle and 80% after two cycles, with no difference across baseline age, line of treatment and MRD burden categories. Toxicity included pyrexia (88%), headache (38%), tremor/chills (29%/25%), nausea/vomiting (22%). Serious adverse events occurred in 5% of the patients (including ataxia/aphasia/encephalopathy).

Blinatumomab was extensively used in Ph− R/R ALL.^59^ In a first exploratory study, 17 out of 25 evaluable patients achieved CR or CRi within two cycles of treatment. Median response duration was 7.1 months and median OS 9.7 months. Three patients relapsed with a CD19 negative clone. A larger confirmatory study was performed in 189 heavily pretreated, high-risk subjects, either primary refractory or in first relapse after a CR lasting <12 months, failing allogeneic SCT or in subsequent relapse.^60^ Forty-three percent of the patients achieved CR/CRi (79% after the first cycle). In responsive patients with evaluable MRD data (n=73), 51 (70%) had a complete MRD response and 9 (n=9) reached an MRD <10^−4^.^61^ Median DFS was 6.9 months in patients with MRD response and 2.3 months in patients without MRD response. Moreover, 40% of responders underwent an allogeneic SCT after blinatumomab only.^62^ The rate of serious adverse events affecting the central nervous system was 2–3%. A final phase III study comparing blinatumomab with standard “investigator choice” chemotherapy in R/R ALL is currently underway (study 311, n=400). Another smaller trial in R/R Ph+ ALL is near to completion in the fall 2014 (study 216, n=41). Of note, in blinatumomab studies some of the relapses occurred at extramedullary sites or were related to the expansion of a CD19− ALL clone.

Beside the several studies in MRD+ and R/R adult ALL, a randomized trial by the ECOG in patients 30–70 years of age (study 1910, n=360) will compare an early consolidation therapy with or without blinatumomab in newly diagnosed Ph− BCP ALL.

The most significant data relative to the use of blinatumomab in B-lineage ALL are summarized in Table 3.

## Modern Immunotherapy with CAR T Cells

### A breakthrough in cellular therapy for BCP ALL

**Figure 3:**
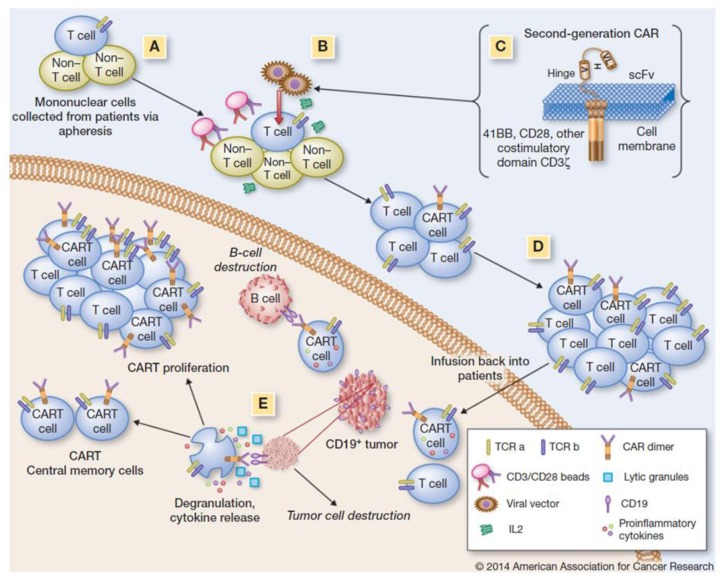
Production and activity of CAR T cells against CD19+ BCP ALL. A, stimulation of T cells using beads coated with CD3/CD28 MoAb’s and with IL2 support (other methods available; see original reference for details). B, transduction of T cells using a viral vector encoding for CD19-CAR (other methods available). C, design of second generation CAR T cells. D, T cell transduction, expansion and differentiation into T efefctor phenotype. E, target recognition, destruction and differentiation into T memory phenotype. (Used with permission, from Kenderian SS, Ruella M, Gill S, Kalos M. Chimeric antigen receptor T cell therapy to target hematological malignancies. Cancer Res. 2014;74:6383–6389).

Normal autologous or allogeneic T cells can be harvested from patients or normal donors to be genetically modified to express a chimeric antigen receptor (CAR) recognizing specific targets on leukemic cells, then expanded and reinfused in the patient to exert antileukemic activity. A CAR consists of a single chain variable antibody fragment highly specific to a tumor antigen, which is fused to the transmembrane domain and a T cell signaling moiety^63^. The resulting receptor, when expressed on the surface of a T cell, mediates binding of the target tumor antigen and activates a signal to the T cell, inducing target cell lysis. Second and third generation CAR T cells present a single-chain variable fragment that resides outside of the T cell membrane and is linked to stimulatory molecules inside the T cell. The general schema for production of CAR T cells and their *in vivo* activity against CD19+ ALL cells is shown in Figure 3.

### Clinical studies with CAR T cells

CAR T cells with specificity for CD19 have shown promising results in chronic lymphocytic leukemia.^64^ Preliminary results of this approach used in two children with R/R ALL were published.^65^ In one case there was a sustained remission. Other recent pre-clinical studies support additional genetic modifications to achieve optimal clinical efficacy.^66^–^68^ Altogether, there is accumulating evidence pointing to the relevant activity of CD19-CAR T cells and CD22-CAR T cells in R/R ALL. These patients are usually prepared with immune suppressive therapy before receiving the CAR T cell infusion (with cyclophosphamide and fludarabine). A breakthrough publication^69^ demonstrated the potential of this treatment in 5 adult patients with R/R ALL (age range 23–66 years). At time of CAR T cell therapy, 3 patients were refractory to salvage chemotherapy, and one was MRD+. After CAR T, all were in clinical and molecular CR, and 4 out of the 5 patients could undergo an allogeneic SCT. Other reports soon followed with either CD19-CAR T or CD22-CAR T, expanding our knowledge about this innovative treatment method.^70^–^73^ Two very recent publications reported the final results of prospective trials using CAR T cells obtained through different methodology on 30 and 21 patients with relapsed ALL, respectively, including a few adult subjects.^74^,^75^ In the first study autologous CD19-CAR T cells induced a CR in 27 (90%) patients (of whom 2 had previously failed blinatumomab, and 15 had relapsed following allogeneic SCT). The event-free survival was 67% at 6 months, associated with persistence of CAR T cells (68%) and B-cell aplasia (73%). In the second trial, aimed at establishing the maximum tolerated dose of CAR T cells (defined as 1×10^6^/kg CAR T cells), the generation of CAR T cells was successful in 20 of 21 patients (90%). Treatment toxicity mediated by cytokine release was fully reversible, prolonged B-aplasia did not occur, and 14 patients got a CR (70%) including 6 of 6 with primary refractory ALL. Moreover, 12 patients achieved MRD negative status, and 10 proceeded to allogeneic SCT. In both studies CAR T cells were detectable in the cerebrospinal fluid, clearing off blast cells in some patients with meningeal leukemia.

Presently, CAR T cell treatment remains experimental and available only at selected centers due to its technical complexity. It is however highly promising and must be developed further as a potential major step forward in the management of adult BCP ALL. CAR T cells carry peculiar toxicities related to cell expansion/activation, resulting in a cytokine-release syndrome which is occasionally associated with cardiorespiratory failure requiring admission to intensive care unit. The interleukin-6 inhibiting agent tocilizumab is effective in this setting. The degree to which this treatment causes a permanent B-cell depletion with severe hypogammaglobulinemia in long-term survivors is another critical point.

## Conclusions

**Figure 4:**
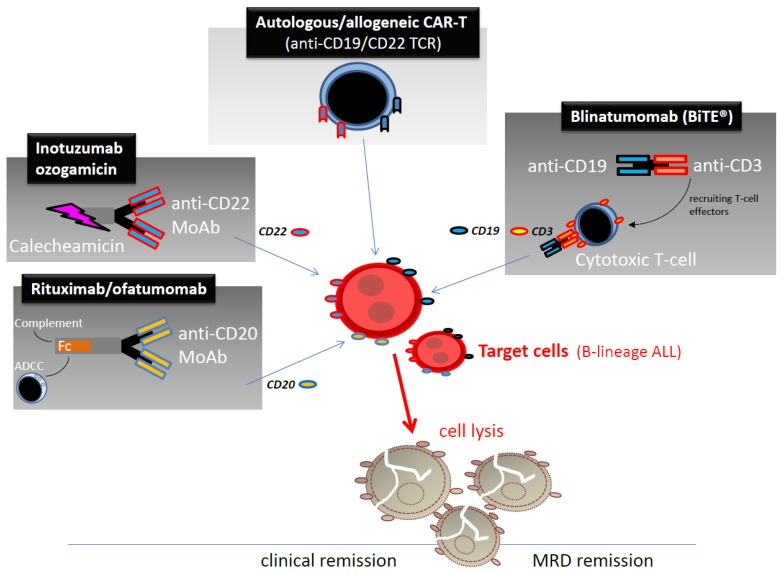
Overview of ALL cell targets and mechanisms of action of rituximab/ofatumomab, inotuzumab ozogamicin, blinatumomab and CAR T cells in adult BCP ALL

Rituximab, IO, blinatumomab and CAR T cells can all contribute through different mechanisms to increase the cure rate in adult B-lineage ALL (Figure 4). Notably, both the clinical effectiveness and the manageable toxicity profile demonstrated by single agent IO and blinatumomab in hundreds of patients with R/R or MRD+ disease make them suitable for immediate evaluation in frontline therapy, with or without associated chemotherapy. The first clinical trials are ongoing. The expectations concern an overall, sound therapeutic advancement compared to current results, as well as a change in the indications for allogeneic SCT in CR1, at least in responsive patients previously defined at high-risk by the persistence of post-induction MRD. As regards CAR T cells, although their use in large scale trials is still precluded by the complexity and cost of the procedure, they could soon become another powerful option to treat this illness, whenever required and beyond the new therapeutic standards set by MoAb/chemotherapy combinations.

## Figures and Tables

**Table 1:** Rituximab in adult ALL: design and results of main clinical studies.

Target antigen expression (CD20)	Study (no. of patients), ref.	Study type and design	Main results (R-treated group/arm vs historical/control group/arm)
40%, BCP ALL (Ph− and Ph+)	MDACC (n=282)^22^	Phase II, frontline therapy (Hyper-CVAD + R), CD20+ Ph− ALL	Age <60 years (n=68): 3-year CR duration 70%, OS 75% (P<.001 and P=.003 vs historical without R) Age >60 years (n=28): 3-year CR duration 45%, OS 28% (P=NS vs historical without R)
	GMALL (n=263)^24^	Phase II, frontline therapy (GMALL 07/2003 + R), CD20+ Ph− ALL	SR (n=196) and HR (n=67); R-treated n=181 (cumulative data) SR group: CR 94%, MRD <10^−4^ at wk 16 90%, 5-year continuous CR 90% and OS 71% with R (without R: 91%, 59%, 47%, 57%) HR group: CR 81%, MRD <10^−4^ at wk 16 64%, 5-year continuous CR of SCT patients 67% and OS 55% (without R: 88%, 40%, 37%, 36%)
100% (Burkitt leukemia/lymphoma)	7 studies (n=604, cumulative)*	Phase II, frontline therapy (various regimens + R), BL	CR 78–100%, 3-year OS 62–100% (average 20% better than historical control)
	GRAALL-Lysa (n=257)^21^	Phase III, frontline therapy (LMBA02 + R vs LMBA02), BL	3-year event-free survival 76% in R arm (n=128) vs 64% in no R arm (n=129, P=0.046)

R, rituximab; NS, not significant; other abbreviations: see text

* Hyper-CVAD (MDACC, n=31), B-NHL 2002 (GMALL, n=185), DA-EPOCH (NCI, n=30), 10 002 (CALGB, n=105), B-NHL 2002 Italy (NILG, n=105), B-NHL 2002 Spain (PETHEMA, n=118), CODOX/M-IVAC (Naples-Turin, n=30)^14^–^20^

**Table 2:** Inotuzumab ozogamicin (IO) in adult ALL: design and results of main clinical studies.

Target antigen expression (CD22)	Study (no. of patients), ref.	Study type and design	Main results
80–90%, BCP ALL (Ph− and Ph+)	MDACC (n=90)^39^	Phase II, R/R ALL, single agent IO (single dose or weekly)	CR 19%, CRi 39%, refractory 38%, early death 4% MRD response (flow-cytometry): 36/50 (72%) Median OS 6.2 months Median CR duration 7 months (42% at 1 year) Allogeneic SCT feasibility: 36 (40%), of whom 13 (36%) alive and well Favorable prognostic factors: treatment at first salvage (median OS 9.2 months), achievement of CR (median survival 13.1 months), MRD negativity (median remission duration 11.5 months)
	MDACC (n=27)^41^	Phase II, frontline therapy of elderly ALL (with mini-Hyper-CVAD)	Patient age 60–79 years (median 69 years) CR 96% MRD response (flow-cytometry): 25/25 CR (100%) 1-year OS 81%

Other abbreviations: see text

**Table 3:** Blinatumomab in adult ALL: design and results of main clinical studies.

Target antigen expression (CD19)	Study (no. of patients), ref.	Study type and design	Main results
98–100%, BCP ALL (Ph− and Ph+)	GMALL (n=21)^56^	Pilot phase II, MRD+ (≥10^−3^) (CR1), single agent blinatumomab	16/20 evaluable patients achieved molecular CR DFS 61% at 2.5 years Superimposable outcome of Ph− MRD responders with (n=6) or without (n=6) allogeneic SCT (DFS 80% at 2.5 years)
	International (n=116)^58^	Confirmatory phase II, MRD+ (≥10^−3^) (CR1), single agent blinatumomab	Median age 45 years (range 18–76 years) 35% MRD+ in second/third CR 88 (78%) had molecular CR after one cycle, 90 after >1 cycle (80%) 79 alive on follow-up No baseline characteristic predictive of MRD response
	GMALL (n=25)^59^	Pilot phase II, R/R Ph− ALL, single agent blinatumomab	17 patients achieving CR/CRi (68%) Median DFS 7.1 months Median OS 9.7 months
	International n=189)^60^	Confirmatory phase II, R/R Ph− ALL, single agent blinatumomab	Median age 39 years (range 18–79 years) 43% overall achieved CR/CRi (79% of whom after 1 cycle) 45% relapsing after allogeneic SCT (n=64) achieved CR/CRi Median DFS 5.9 months MRD response in evaluable CR/CRi patients (n=73): 80% (70% complete) Median OS for MRD responders 11.4 months (vs 6.7 months) Median DFS for MRD responders 6.9 months (vs 2.3 months)

Other abbreviations: see text
